# Hyperspectral reflectance as a tool to measure biochemical and physiological traits in wheat

**DOI:** 10.1093/jxb/erx421

**Published:** 2017-12-22

**Authors:** Viridiana Silva-Perez, Gemma Molero, Shawn P Serbin, Anthony G Condon, Matthew P Reynolds, Robert T Furbank, John R Evans

**Affiliations:** 1 CSIRO Agriculture, Canberra, ACT, Australia; 2 ARC Centre of Excellence for Translational Photosynthesis, Research School of Biology, The Australian National University, Canberra, ACT, Australia; 3 Environmental, and Climate Sciences Department, Brookhaven National Laboratory, Upton, NY, USA; 4 International Maize and Wheat Improvement Centre (CIMMYT), México, DF, Mexico

**Keywords:** Electron transport rate, hyperspectral reflectance, leaf dry mass per area, leaf nitrogen, partial least squares, photosynthesis, Rubisco, *Triticum aestivum*, velocity of carboxylation

## Abstract

Improving photosynthesis to raise wheat yield potential has emerged as a major target for wheat physiologists. Photosynthesis-related traits, such as nitrogen per unit leaf area (N_area_) and leaf dry mass per area (LMA), require laborious, destructive, laboratory-based methods, while physiological traits underpinning photosynthetic capacity, such as maximum Rubisco activity normalized to 25 °C (*V*_cmax25_) and electron transport rate (*J*), require time-consuming gas exchange measurements. The aim of this study was to assess whether hyperspectral reflectance (350–2500 nm) can be used to rapidly estimate these traits on intact wheat leaves. Predictive models were constructed using gas exchange and hyperspectral reflectance data from 76 genotypes grown in glasshouses with different nitrogen levels and/or in the field under yield potential conditions. Models were developed using half of the observed data with the remainder used for validation, yielding correlation coefficients (*R*^2^ values) of 0.62 for *V*_cmax25_, 0.7 for *J*, 0.81 for SPAD, 0.89 for LMA, and 0.93 for N_area_, with bias <0.7%. The models were tested on elite lines and landraces that had not been used to create the models. The bias varied between −2.3% and −5.5% while relative error of prediction was similar for SPAD but slightly greater for LMA and N_area_.

## Teaser

Leaf hyperspectral reflectance can be used by the wheat physiology and breeding communities to rapidly estimate Rubisco activity, electron transport rate, leaf nitrogen, leaf dry mass per area, and relative chlorophyll content.

## Introduction

Global population is predicted to reach 9.7 billion by 2050 (UN Department of Economic and Social Affairs, 2015). To satisfy projected demand for cereal grain, wheat yields need to increase at rates far exceeding the current annual genetic gains being made in most parts of the world by plant breeders (Reynolds *et al.*, 2012). Further improvements in yield require increases in biomass, derived from improvements in radiation use efficiency and photosynthetic traits (Parry *et al.*, 2011; Reynolds *et al.*, 2012). Despite its importance, selection based on physiological and biochemical characteristics of wheat genotypes in a breeding programme is uncommon due to cost and the time required for testing at a breeding scale. The development of tools that improve speed and accuracy of estimating biomass and photosynthesis-related traits would allow screening of a large number of lines, making these traits more amenable to incorporation into breeding programmes. This would also facilitate identification of molecular markers and candidate genes underpinning genetic variation for the traits of interest. Spectral reflectance is associated with specific plant characteristics and has been proposed as a fast and non-destructive technique that can be efficiently used in breeding programmes where thousands of individuals must be screened every year (Babar *et al.*, 2006).

Prediction of photosynthesis-related traits through simple leaf reflectance parameters is well established. Reflectance in the visible/near infrared part of the electromagnetic spectrum has been related to xanthophylls, chlorophylls, and water in plants, and the red edge in the derivative of reflectance is commonly related to photosynthesis (Peñuelas and Filella, 1998). One of the first and most widely used optical instruments is the SPAD chlorophyll meter. This measures transmittance of red (650 nm) *versus* infrared (940 nm) light to estimate leaf chlorophyll content (Benedict and Swidler, 1961; Inada, 1963; Mullan and Mullan, 2012). Numerous indices based on wavelengths in the visible and infrared part of the electromagnetic spectrum have been used in remote sensing to predict vegetation biomass, biochemical leaf components and some physiological traits. For example, the normalized difference vegetative index is used to monitor vegetation using red, infrared and near-infrared wavelengths to measure relative greenness, foliage development, senescence, biomass, and chlorophyll content (Tucker, 1979; Goward *et al.*, 1985; Gamon *et al.*, 1995; Cabrera-Bosquet *et al.*, 2011; Lopes and Reynolds, 2012; Pinto *et al.*, 2016). The water index is used to infer water content from reflectance ratios between 900 and 970 nm (Peñuelas *et al.*, 1997) while the photochemical reflectance index at 531 and 570 nm has been used to estimate radiation-use efficiency and photoprotective pigment pools in leaves (Gamon *et al.*, 1992; Peñuelas *et al.*, 2011).

The infrared (IR) part of the spectrum is commonly divided in to three regions: near infrared (770–1300), short wave infrared 1 (SWIR1; 1300–1900 nm), and short wave infrared 2 (SWIR2; 1900–2500 nm). Research in the IR has increased because hyperspectral cameras and field spectroradiometers are increasingly able to accurately measure the full spectrum (i.e. 350–2500 nm) and because the incorporation of information from the entire visible to SWIR2 region has proven useful for a range of plant traits (e.g. Singh *et al.*, 2015; Yang *et al.*, 2016). IR spectra measured from leaves have been correlated with photosynthetic parameters (maximum Rubisco activity, *V*_cmax_, and electron transport rate, *J*; Serbin *et al.*, 2012; Ainsworth *et al.*, 2014), and have been used to predict carbon, nitrogen, and phosphorus content of leaves (Gillon *et al.*, 1999). Successful predictions of photosynthetic parameters have been obtained for tropical trees, aspen, cotton, soybean, and maize (Doughty *et al.*, 2011; Serbin *et al.*, 2012; Ainsworth *et al.*, 2014; Yendrek *et al.*, 2017), and nitrogen content and leaf dry mass per area (LMA) in wheat (Ecarnot *et al.*, 2013). In wheat at the canopy level, predictions from hyperspectral reflectance for biomass, nitrogen, and water content have been demonstrated (Hansen and Schjoerring, 2003; Pimstein *et al.*, 2007; Yao *et al.*, 2015). These examples show the potential of using hyperspectral reflectance to screen wheat for photosynthetic parameters (Garriga *et al.*, 2017).

The main objective of this study was to develop statistical models linking leaf-level hyperspectral reflectance to photosynthetic traits, thereby establishing a high throughput alternative to the traditional time-consuming methods. Leaf reflectance spectra are correlated with photosynthetic traits derived from the response of CO_2_ assimilation to CO_2_ concentration using the model of Farquhar *et al.* (1980) considering the new parameters for wheat (Silva-Pérez *et al.*, 2017). The method is validated for *V*_cmax_, *J*, and with LMA, N_area_ and SPAD (a surrogate for chlorophyll content). Examples are given where the derived models are used to predict SPAD, LMA and N_area_ in two previously unseen sets of elite and landrace wheat genotypes.

## Materials and methods

### Plant material

Six sets of diverse wheat (*Triticum aestivum*, *T. turgidum*) and triticale germplasm were used in these experiments as follows: (i) Early Vigour (EV): 16 wheat genotypes from CSIRO in Australia, most of which have a larger embryo, fast leaf area development, and low leaf mass per unit area; (ii) a subset of the Best and Unreleased Yield Potential (BYP): 21 wheat genotypes and nine triticale genotypes with high yield in Australia; (iii) CIMMYT Core Germplasm Subset II (C): 30 wheat genotypes selected at CIMMYT (International Maize and Wheat Improvement Center) for high yield (González-Navarro *et al.*, 2015); (iv) Candidates of C (CC): 216 elite wheat genotypes plus seven wheat genotypes from C, in total giving 223 wheat genotypes; (v) wheat landraces (L) obtained from CIMMYT’s gene bank: 230 wheat landraces plus five elite wheat genotypes including two from CC, giving 235 wheat genotypes in total; and (vi) a subset of L (LS): 23 genotypes with similar phenology. An additional letter added to each abbreviation indicates whether the measurements were made before anthesis (B) or at anthesis (A).

### Experimental conditions

The Zadoks scale was used to describe the growth stages (GS) of wheat (Zadoks *et al.*, 1974). The first day after emergence (DAE) is considered at GS10, when at least 50% of the first leaves emerging through coleoptile are visible. Five experiments were conducted: Aus1, Aus2, Aus3, Mex1, Mex2 (Table 1), as follows. 

**Table 1.** Summary of experiments Aus1, glasshouse experiment, CSIRO Black Mountain, Australia (2012); Aus2, glasshouse experiment, CSIRO Black Mountain, Australia (2012); Aus3, field experiment, GES-CSIRO, Australia (2013); Mex1, field experiment, CENEB-CIMMYT, Mexico (2012–2013); Mex2, field experiment, CENEB-CIMMYT, Mexico (2012–2013); stage A, anthesis; stage B, booting (before anthesis); DAE: days after emergence.

Expt	Set of genotypes	Genotypes (repetitions)	Stage (DAE)	Traits
Aus1	EVA(−N), (+N)	16 (3)	A (73–83)	*V*_cmax25_, *J* SPAD, N_mass_, N_area_, LMA, P_mass_, P_area_
Aus2	BYPB (−N), (+N)	30 (2)	B (48–56)	*V*_cmax25_, *J* SPAD, N_mass_,N_area_, LMA, P_mass_, P_area_
Aus3	BYPB	28 (4)	B (46–54)	*V*_cmax25_, *J* SPAD, N_mass_, N_area_, LMA, P_mass_, P_area_
	EVA	2 (4)	A (62–67)	*V*_cmax25_, *J* SPAD, N_mass_, N_area_, LMA, P_mass_, P_area_
	CA	21 (4)	A (60–67)	*V*_cmax25_, *J* SPAD, N_area_, LMA
Mex1	CB	30 (3)	B (67–82)	SPAD, N_mass_
	CA	30 (3)	A (88–103)	*V*_cmax25_, *J* SPAD, N_area_, LMA
Mex2	CC	223 (2)	A (101–103)	SPAD
	L	230 landraces 40 elite wheat	A (110–111)	SPAD
	LS	23 landraces 2 elite wheat	A (117)	N_area_, LMA

The first glasshouse experiment, Aus1, was set up at CSIRO Black Mountain, Canberra, Australia (−35.271875, 149.113982). Two seeds of the EVA set were sown in cylindrical pots of 1.06 litres (15 × 5 cm) with 75:25 loam:vermiculite containing basal fertilizer, and one plant per pot was kept for the experiment. Plant emergence was on 8 April 2012; artificial light was used in June to extend the photoperiod to 16 h; and temperature was controlled to 25/15 °C (day/night). Aus1 was designed to achieve a range in leaf colour with nitrogen deficiency in one treatment (−N) and high fertilizer in the other treatment (+N), and the experiment was organized in a randomized block design, three blocks representing each repetition for +N and other three blocks −N. Extra fertilizer (Thrive, ~300 ml per pot of 1.77 g l^−1^; 27% N, 5.5% P, 9% K) was applied each week for the +N treatment until 83 DAE. A severe low nitrogen treatment was obtained irrigating the pots with water without fertilizer 1.5 months before measurements. The flag leaf was measured at the end of booting and during anthesis (GS58–69) from 73 to 83 DAE.

The second glasshouse experiment, Aus2, was carried out at CSIRO Black Mountain, Canberra, Australia. Three seeds of the BYPB set were sown in pots of 5 litres with 75:25 loam:vermiculite soil mix containing basal fertilizer, and two plants per pot were kept for the experiment. Plant emergence was on 17 October 2012 and temperature was controlled to 25/15 °C (day/night). Aus2 was organized in a randomized block design, two blocks representing each repetition for the high nitrogen treatment (+N) and one block for the low nitrogen treatment (−N). For the +N treatments extra fertilizer (Aquasol, ~300 ml per pot of 1.77 g l^−1^; 23% N, 4% P, 18% K) was applied every 3 d from 41 to 56 DAE. Treatment −N was obtained irrigating the plants with water without fertilizer 10 d before measurements. Treatment −N was applied over a shorter duration than Aus1, resulting in smaller differences in leaf nitrogen content per unit leaf area and photosynthetic parameters. The flag leaf was measured before anthesis (GS49–57) from 48 to 56 DAE.

Experiment Aus3 was carried out in the field at CSIRO Experimental Station at Ginninderra, Australia (−35.199837, 149.090898). The emergence of plants was on 4 October 2013. From 1 to 75 DAE the average maximum for daily temperature (see Supplementary Fig. S1 at *JXB* online) was 22.4 °C and the minimum 7.7 °C, with in total 142 mm of rain and an accumulative thermal time of 1126.8 °C d (base temperature 0 °C). Average solar radiation was 24 MJ m^−2^ (Supplementary Fig. S1). Due to late sowing and long days (~11 h) the wheat cycle was short. The CA and EVA subsets of wheat genotypes were sown in the same experimental design of two randomized blocks. Each block was subdivided into 30 plots (5 × 6). Next to this experimental design, another experimental design of two randomized blocks for the BYPB collection was sown. In this case, each block was subdivided into 42 plots (7 × 6). Each plot for both experimental designs was 5 m×1.8 m. It contained a single genotype sown in 10 rows, 18 cm apart, and approximately 200 plants m^−2^. Plots were fertilized and irrigated optimally in all conditions. For the BYPB subset of wheat genotypes, the flag leaf was measured before anthesis (GS40–55, 46–54 DAE) where the maximum and minimum temperatures were 28.3 and 5.4 °C, respectively. The maximum and minimum temperatures during measurement of EVA (GS69, 62–67 DAE) and CA (GS56-69, 60–67 DAE) were 32.2 and 4.3 °C, respectively. Measurements and sampling were done twice in two plots, resulting in four repetitions for four to five genotypes per day that were at similar plant stage. Due to the close phenology among the lines studied, the number of genotypes measured was reduced: two wheat genotypes from EVA, 20 wheat genotypes and six triticale genotypes from BYPB, and 22 wheat genotypes from CA.

Experiment Mex1 was carried out in the field at Centro Experimental Norman E. Borlaug (CENEB) research station, located in the Yaqui Valley, Sonora, Mexico (27.370837, −109.930362) for a winter–spring cycle. Plant emergence was on 2 December 2012. From the 1 to 138 DAE, the average maximum and minimum daily temperatures were 26 and 8.3 °C, respectively (see Supplementary Fig. S1). In total, 15.4 mm of rain was supplemented with 500 mm of irrigation delivered over five events. The cumulative thermal time was 2364.6 °C d and average daily solar radiation was 17 MJ m^−2^ (see Supplementary Fig. S1). Plants were organized in a randomized 5 × 6 lattice experimental design with three repetitions. Each repetition (10 × 3 plots) enclosed two subdivisions of 5 × 3 plots. Each plot (2.4 m×8.5 m) contained a single genotype sown in six rows, two beds in the middle with two rows each and two beds in the edges with one row of the same genotype, the second row in the edges corresponded to the next genotype or a filling genotype to avoid border effect. Beds followed the system 56–24, where 56 cm is the furrow width and 24 cm is the raised bed width. Plants were grown under optimal management in the field. First fertilization was at soil preparation with 50 kg ha^−1^ of N and 50 kg ha^−1^ of P and a second fertilization in the first irrigation of 150 kg ha^−1^ of N. For the CB subset of wheat genotypes, the flag leaf was measured before anthesis (GS49–57, 67–82 DAE), with maximum and minimum temperatures of 29.7 and 1.5 °C, respectively. For the CA subset, flag leaves were measured at anthesis (GS65 + 7, 88–103 DAE), with maximum and minimum temperatures of 32.1 and 2.5 °C, respectively. Measurements and sampling were from one plant per plot; three to six genotypes per day were measured at a similar plant stage with three repetitions.

Field experiment Mex2 was used to test the reflectance method developed in this study with a larger, diverse group of wheat genotypes. CC and L genotypes were sown at the same time and near the plots from the Mex1 experiment at CENEB during the same season with the same sowing and plant emergence dates and crop management and weather (see Supplementary Fig. S1). Plots in both sets of wheat genotypes were 2 m long×1.6 m, and each one contained two beds arranged in the 56–24 system. CC plants were arranged in the field in 20 × 22 plots plus six plots in the 23rd row of plots to give 446 plots in total, and the whole experiment comprised two randomized blocks. L plants were sown in a band of 5 × 54 plots. From these 270 plots, 230 plots contained single landrace wheat genotypes and 40 plots contained elite wheats (checks), placed after every tenth landrace plot. The measurements were done in two main steps as follows. (i) Survey: CC and L flag leaves were measured for reflectance and SPAD on all plots including repetitions and checks. CC (*n*=446) plants were measured from 101 to 103 DAE, which was 15 d after anthesis on average. L plants (*n*=270) were measured from 110 to 111 DAE, which varied from 1 to 36 d after anthesis (Supplementary Fig. S2). (ii) Second measurement: a selection of 23 L genotypes that were 5–10 d after anthesis were identified (Supplementary Fig. S2) and measured a second time (LS). Reflectance and SPAD were measured and leaves were sampled for determination of LMA and N_area_.

### Measured traits

Gas exchange was measured using a LI-COR LI-6400XT infrared gas analyser (LI-COR Inc., Lincoln, NE, USA); the 6 cm^2^ rectangular head was used for the experiments Aus1, Aus2, and Aus3, and the 2 cm^2^ circular fluorescence head (Li-6400–40; LI-COR Inc.) for the Mex1 experiments. The flow rate into the leaf CO_2_ chamber of the Li-COR was set at 500 μmol s^−1^ for the 6 cm^2^ head and 350 μmol s^−1^ for the 2 cm^2^ head, irradiance was 1800 μmol quanta m^−2^ s^−1^, and block temperature was 25 °C. Gas exchange was used to measure the rate of CO_2_ assimilation (*A*) and stomatal conductance (*g*_s_) at 400 inlet μmol CO_2_ mol^−1^ initially followed by a CO_2_ response curve (inlet CO_2_ concentrations are shown in Supplementary Table S1). The maximum Rubisco activity normalized to 25 °C, *V*_cmax25_, and electron transport rate, *J*, were calculated using the leaf biochemical model of photosynthesis (Farquhar *et al.*, 1980) with kinetic constants derived for wheat (Silva-Pérez *et al.*, 2017).

Flag leaves were measured with a SPAD-502 chlorophyll meter (Minolta Camera Co., Ltd, Japan) to provide a non-destructive surrogate for chlorophyll content (Mullan and Mullan, 2012). In all experiments, three SPAD readings taken from the same region of the leaf used for leaf reflectance and gas exchange measurements were averaged per leaf.

Following gas exchange experiments in Aus1, Aus2, and Aus3, leaf material was sampled 3 cm up and down the leaf from where the chamber was clipped on in order to determine leaf mass per unit area (LMA) and nitrogen concentration. Area of the leaf samples was calculated from a digital photo using the program ImageJ v1.47. Samples were then dried for 48 h at 70 °C to achieve constant mass and weighed on an analytical balance (Mettler Toledo, AT201, 0.01 mg) to obtain LMA (g m^−2^). Leaf nitrogen concentration (N_mass_; mg g^−1^) and phosphorus concentration (P_mass_; mg g^−1^), were determined on the same samples by flow injection analysis (QuikChem^®^ method, Lachat Instruments, CO, USA) after Kjeldahl digestion of leaves. For Mex1 and LS-Mex2 experiments, a complete flag leaf was measured using a leaf area meter (LI3050A/4; LI-COR), followed by drying for 48 h at 70 °C and weighing on a precision balance (Ohaus Adventurer, AR1530, 0.001 g) to obtain LMA. N_mass_ was determined at CIMMYT Batan, Mexico with the Technicon AutoAnalyzer II (Galicia *et al.*, 2008). N_mass_ or P_mass_ and LMA were used to calculate nitrogen content per unit leaf area (N_area_; g m^−2^) and phosphorous content per unit leaf area (P_area_; g m^−2^).

### Reflectance measurements

Reflectance spectra were measured with a FieldSpec®3 (Analytical Spectral Devices, Boulder, CO, USA) full range spectroradiometer (350–2500 nm) coupled via the fibre optic cable to a leaf clip with an internal calibrated light source and with two panels, a white panel used for instrument calibration and a black panel used for measurements (Analytical Spectral Devices, Boulder, CO, USA). The calibration (i.e. white reference) of 100 reflectance spectra took 20 s and the leaf measurement took a maximum of 30 s in the Aus1 experiment. At this stage, reflectance was measured using two pieces of leaf measured in the horizontal position (Supplementary Fig. S3A). The technique was improved in the Aus2, Aus3, Mex1, and Mex2 experiments, where the calibration of 30 reflectance spectra took 6 s and the leaf measurement took 9 s, with each leaf placed vertically, which helped to speed up the measurements in the field (Supplementary Fig. S3B). In these experiments a mask was used to reduce the leaf-clip aperture to an elliptic area of 1.264 cm^2^ (1.15 × 1.4 cm) suitable for wheat leaves, a black circular gasket of 2.2 cm inner diameter and 3 mm thickness was pasted to the mask to avoid leaf damage and to eliminate potential entry of external light through the edges (Supplementary Fig. S3C). In experiments Aus1, Mex1, and Mex2, one reflectance measurement was made per leaf lamina, two in Aus2, and three in Aus3, which were averaged. The leaf lamina repetitions are independent from the experimental design repetitions.

### Analysis of leaf reflectance spectra

Leaf spectra required pre-treatment to correct for the ‘jump’ observed in apparent reflectance when changing between the detectors. First, two different jump corrections were applied to the reflectance measurements because two different ASD FieldSpec®3 spectroradiometers were used, one in Australia and the other in Mexico. Reflectance measured with the FieldSpec3 in Australia was corrected at 1000 and 1800 nm. Reflectance measured with the FieldSpec3 in Mexico was corrected at 1000 and 1830 nm using the software Spectral Analysis and Management System (SAMS®), version 3.2. Spectra with reflectance lower than 0.35 and higher than 0.6 at 800 nm were removed because an earlier analysis had shown these to be outliers. Finally, only the spectrum from 400 to 2400 nm was used in the analysis.

**Fig. 1.**
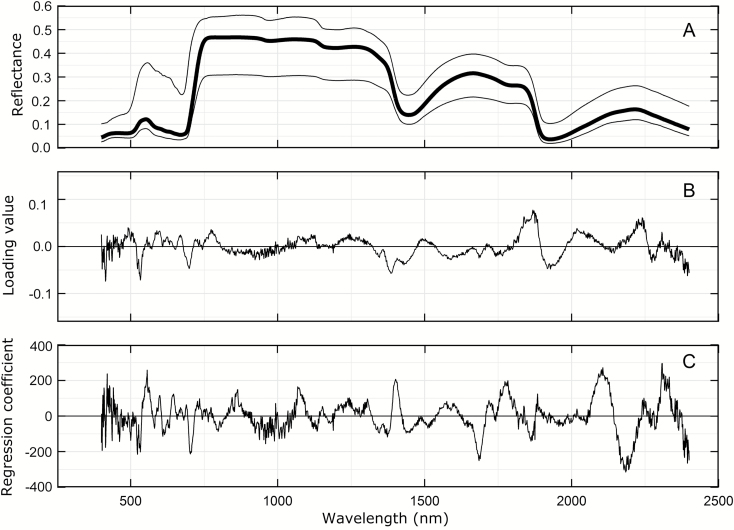
(A) Reflectance from Aus1, Aus2, Aus3, and Mex1 experiments (*n*=565) from 400 to 2400 nm. The bold line is the mean and the range is given by the upper and lower lines. (B) Loadings and (C) regression coefficients of the model for *V*_cmax25_ with 18 components.

Analysis of the reflectance data was performed using the pls package Principal Component and Partial Least Squares Regression in R (Mevik and Wehrens, 2007) under R software version 2.15.0. One or two repetitions from experiments Aus1, Aus2, Aus3, and Mex1 were used as training data (about 55% of the total observed data) to ensure that the complete set of genotypes were present in both training and test data (see Supplementary Table S2). The remaining repetitions from experiments Aus1, Aus2, Aus3, and Mex1 were used only as test data (about 45% of the observed data) to validate the partial least squares regression (PLSR) models. The number of components used in the regression model fitted to the reflectance data was based on the smallest root mean square error of the cross validation (RMSEP-CV) and the smallest predicted residual sum of squares (PRESS) from the training data. PLSR generates loadings and scores that are used to generate a group of regression coefficients for each wavelength and an intercept, which we call the PLSR model. The PLSR model is different for each trait (Supplementary Fig. S4). An example of the reflectance measurements, loadings and regression coefficients for 18 components obtained for *V*_cmax25_ is shown in Fig. 1.

Evaluation of the model accuracy included the coefficient of determination (*R*^2^), the model bias:

$$\text{Bias}\;(\%)=100\times (\bar{\hat{y}}-\bar{y})/\bar{y}$$ (1)

to represent the percentage of the difference between the mean of the predicted trait, $\bar{\hat{y}}$, and the mean of the observed trait, $\bar{y}$, and the relative error of prediction (REP) (Nguyen and Lee, 2006):

$$\text{REP}\;(\%)=100\times {\left[\frac{1}{n}\overset{n}{\underset{i=1}{\sum }}{\left(y_{i}-{\hat{y}}_{i}\right)}^{2}\right]}^{0.5}/\bar{y}$$ (2)

to represent the percentage of the root mean square error in prediction, where $y_{i}$ and ${\hat{y}}_{i}$ are observed and predicted traits, *n* is the number of sample in data set and $\bar{y}$ is the mean of the observed values of traits.

### Applying the PLSR models

One objective of this study was to assess whether leaf-level hyperspectral reflectance could be used as a high throughput alternative to traditional and time-consuming measurements of destructive analyses for biomass-related and photosynthetic traits. Experiment Mex2 included 458 elite wheat genotypes (CC-Mex2) and landraces (L-Mex2) (Table 1) that were independent from the genotypes used to train and validate the models. They were surveyed with hyperspectral leaf reflectance and SPAD. At the time the wheat landraces were surveyed for leaf reflectance, their phenological development ranged from 7 d before to 36 d after anthesis (see Supplementary Fig. S4). Consequently, 21 wheat landraces and two elite wheats (checks) between 6 and 9 d after anthesis were selected for the LS-Mex2 experiment, where hyperspectral leaf reflectance was measured and leaves were sampled to obtain LMA and N_area_.

## Results

### Predictions and validation of traits

**Fig. 2.**
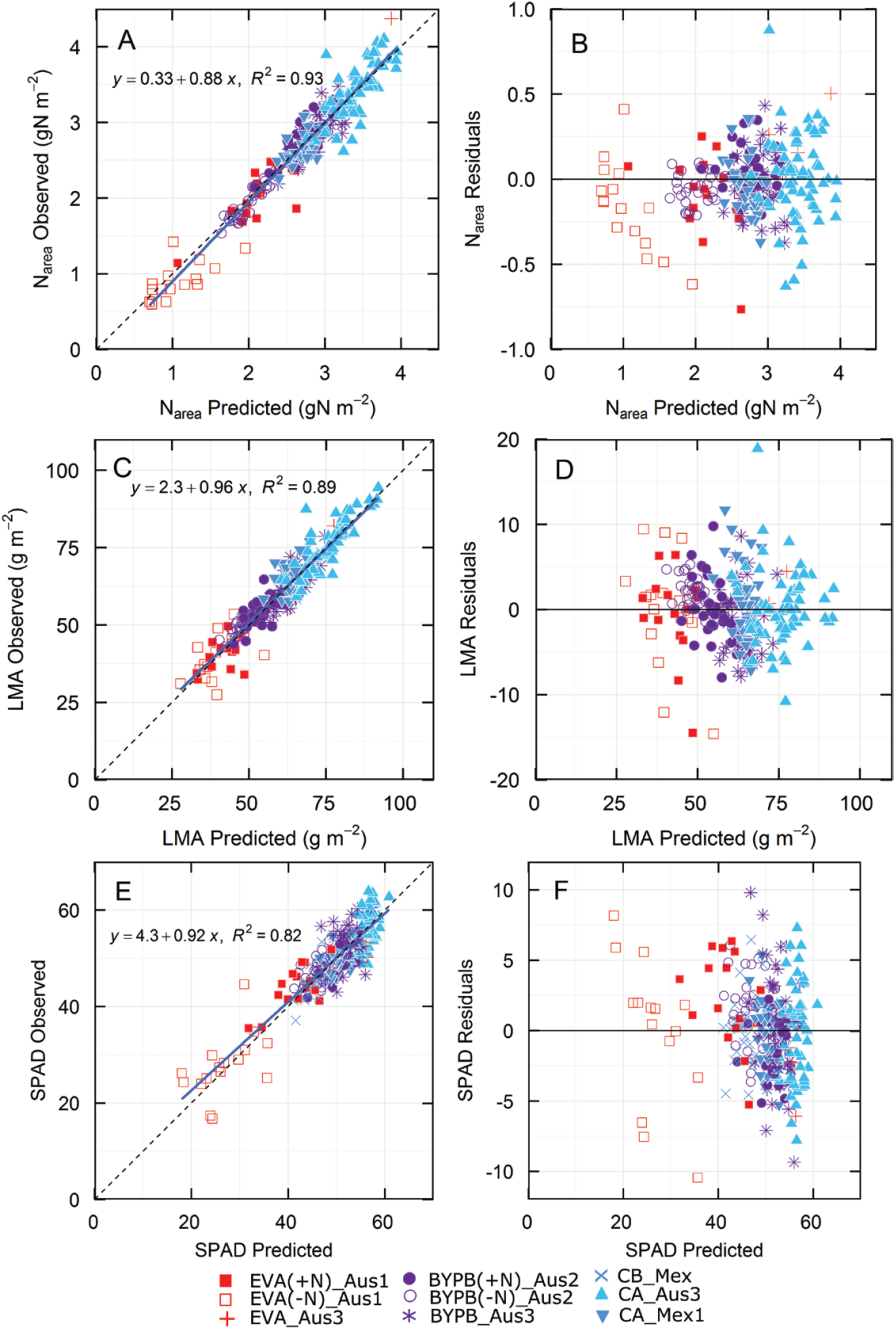
Validation of predictions (A, C, E) and residuals (B, D, F) for N_area_ (21 components), LMA (21 components), and SPAD (16 components). Symbols show only the validation data, i.e. those that were not used to construct the models. See Table 2 for details. (This figure is available in color at *JXB* online.)

Predictions for N_area_, LMA, and SPAD had higher coefficients of determination than for the photosynthetic parameters and observations followed the 1:1 line (Fig. 2; bias <0.7%, Table 2). For these traits, the residuals were smaller and showed no underlying trends. N_mass_ had a smaller coefficient of determination than N_area_ (*R*^2^=0.7 *vs* 0.93; Table 2).

**Table 2.** Statistical parameters of the PLSR model validation data set The lowest RMSEP-CV was used to choose the number of components in the model. NC, number of components; REP, relative error of prediction; RMSEP CV, root mean square error of prediction from cross validation with PLSR; Tr, training set; Val, validation or test data.

Traits	N Tr	N Val	RMSEP CV	NC	R^2^ Tr	R^2^ Val	REP Val (%)	Bias Val (%)
N_area_	282	243	0.22	21	0.92	0.93	7.6	0.73
LMA	282	243	4.50	21	0.86	0.89	7.0	-0.23
SPAD	342	272	3.16	16	0.87	0.81	6.8	-0.34
*V*_cmax_	262	226	31.53	23	0.79	0.74	18.7	0.20
*J*	262	226	25.44	18	0.82	0.70	13.0	-0.73
N_mass_	342	273	3.30	24	0.86	0.70	10.5	1.3
P_mass_	219	212	0.93	19	0.54	0.65	25.8	3.3
*V*_cmax25_	262	226	20.68	18	0.76	0.62	15.9	0.17
*A*	307	253	3.93	15	0.64	0.49	17.7	0.49
*V*_cmax25_/N_area_	262	226	10.62	14	0.40	0.48	17.5	1.9
P_area_	219	212	0.04	19	0.40	0.42	23.5	4.2
*g*_s_	307	253	0.15	11	0.50	0.34	33.5	3.3

**Fig. 3.**
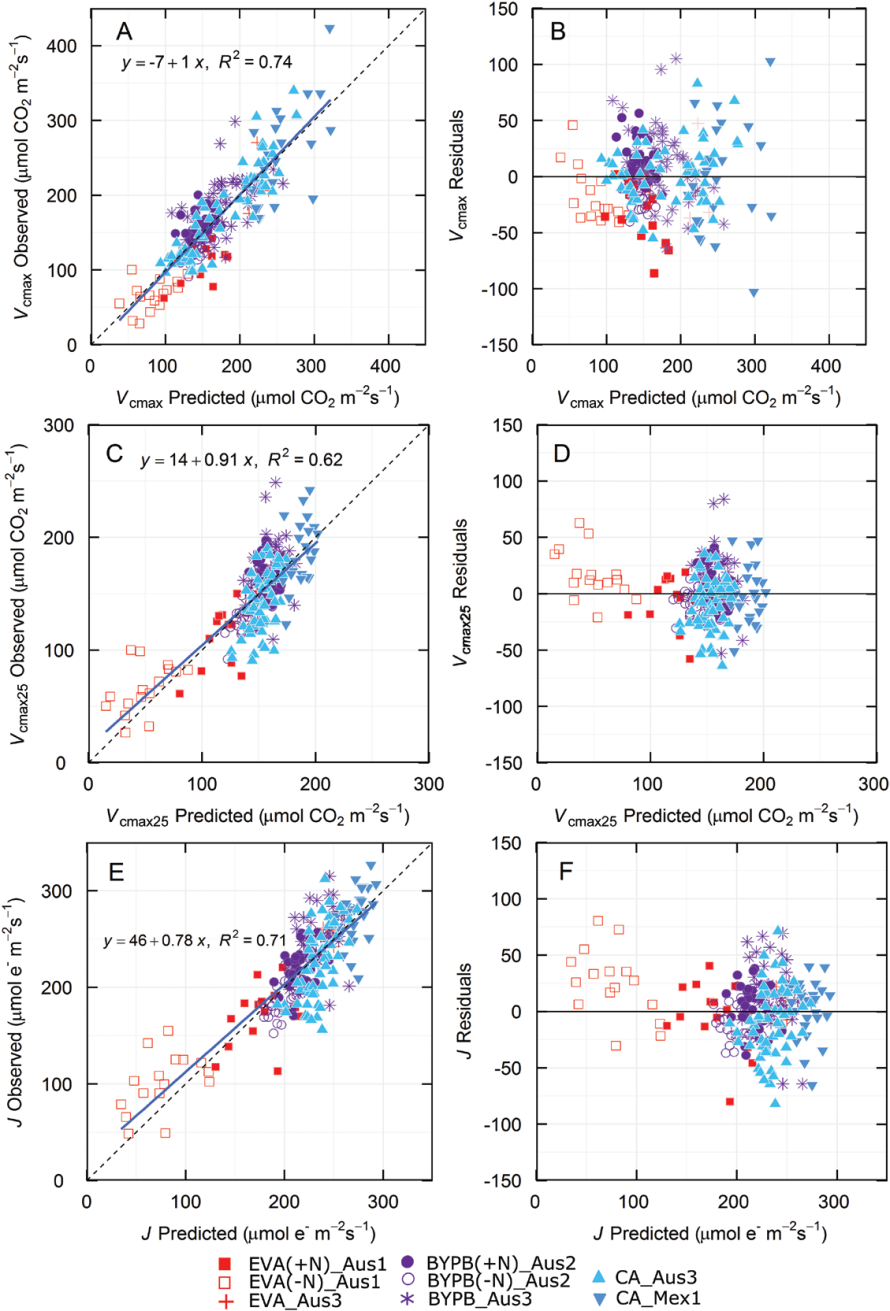
Validation of predictions (A, C, E) and residuals (B, D, F) for *V*_cmax_ (23 components), *V*_cmax25_ (18 components) and *J* (18 components). Symbols show only the validation data, i.e. those that were not used to construct the models. See Table 2 for details. (This figure is available in color at *JXB* online.)

Two predictions are shown for the Rubisco-related trait *V*_cmax_: (i) *V*_cmax_ without leaf temperature correction and (ii) *V*_cmax25_ corrected to a common leaf temperature of 25 °C using *in vivo* Rubisco kinetics derived for wheat (Silva-Pérez *et al.*, 2017). Both predictions fell approximately on the 1:1 line (Fig. 3; bias <0.2%). The residuals between observed data and predictions were larger for *V*_cmax_ than *V*_cmax25_.

In the case of *J*, predictions fell about the 1:1 line with the coefficient of determination (*R*^2^=0.71) slightly less than for *V*_cmax_ (*R*^2^=0.74; Fig. 2). The trends of *J* predictions and residuals are similar to *V*_cmax25_.

When Kjeldahl digestion was used to determine leaf nitrogen, we also obtained a measure of phosphorus. Predictions of leaf phosphorus from hyperspectral reflectance were not as good as for nitrogen (P_mass_, *R*^2^=0.65; P_area_, *R*^2^=0.42; Table 2).

### Predicting *V*_cmax25_/N_area_

**Fig. 4.**
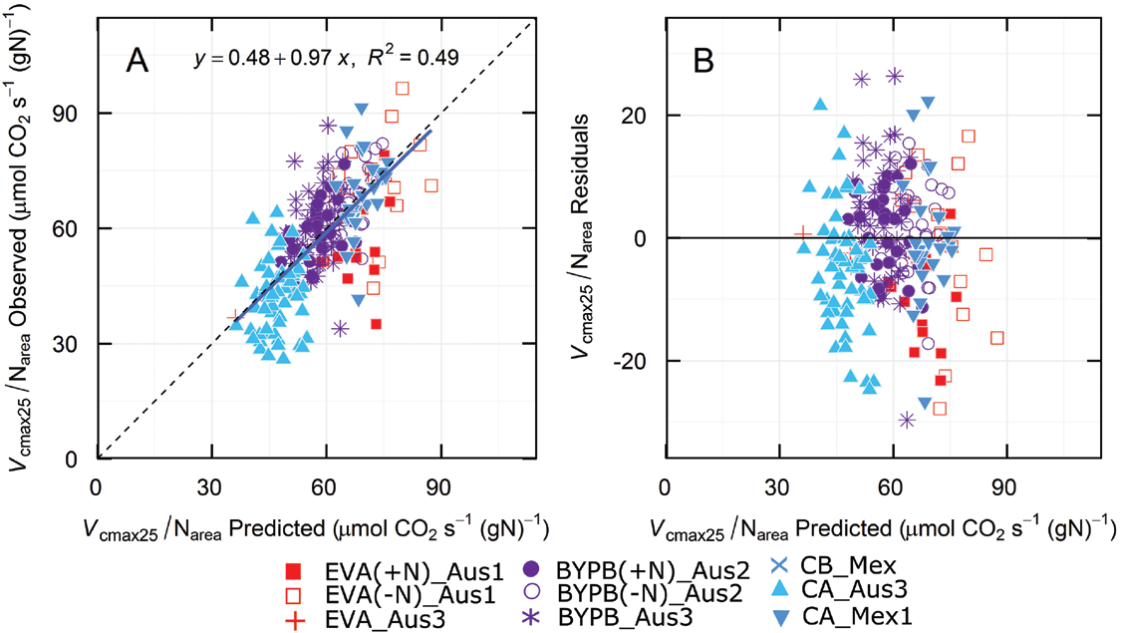
(A) Validation of predictions and (B) residuals for *V*_cmax25_/N_area_ (13 components). Symbols show only the validation data, i.e. those that were not used to construct the models. See Table 2 for details. (This figure is available in color at *JXB* online.)

Given the fact that CO_2_ assimilation rate, *A*, and stomatal conductance, *g*_s_, are variable for a given leaf and depend on environmental conditions, it was not surprising that their prediction was generally low (*A*, *R*^2^=0.49; *g*_s_, *R*^2^=0.34; Table 2). Instead, we targeted underlying photosynthetic capacity normalized per unit leaf nitrogen, *V*_cmax25_/N_area_. For this trait, which represents photosynthetic efficiency (Rubisco capacity per unit leaf N), the model predictions fell about the 1:1 line (*R*^2^=0.49; bias 1.9%; Fig. 4). Interestingly, the coefficient of determination for *V*_cmax25_/N_area_ predicted as a ratio was greater than when the trait was calculated from the ratio of values of *V*_cmax25_ and N_area_ predicted separately (*R*^2^=0.13).

In general, the residuals showed no underlying trends when plotted against the predicted data (Figs 2–4). However, there was a positive trend within each experimental group when residuals were plotted against observed data (see Supplementary Fig. S5).

### Predicting traits for novel wheat genotypes that were not used for PLSR model derivation

**Fig. 5.**
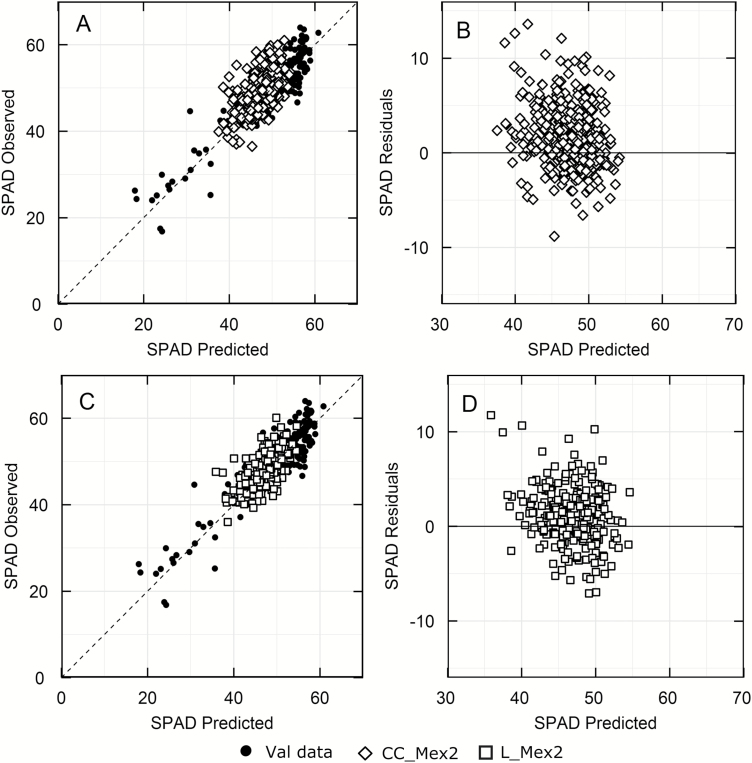
Comparison of SPAD predicted from reflectance using the model developed in this study (Supplementary Fig. S4) and actual SPAD measurements for elite wheat (CC-Mex2, open diamonds, A, B) or the wheat landraces set (L-Mex2, open squares, C, D) and with their respective residuals (B, D). The dashed line represents the 1:1. CC, *n*=448, L, *n*=270 and Val data, *n*=272. Closed circles are the validation data from Fig. 2E.

To assess the use of hyperspectral reflectance as a high throughput tool in the field, 458 elite wheat genotypes and landraces (Mex2) were surveyed. The predicted values of SPAD fell about the 1:1 line and the relative error of prediction for SPAD compared favourably to that observed for the validation data (CC-Mex2 7.4% and L-Mex2 6.6%; Table 3; cf. 6.8%, Table 2). The distribution of the residuals showed no underlying trend (Fig. 5B, D) and it was similar to that observed with the validation data (see Supplementary Fig. S6A, B).

**Table 3.** Statistical parameters assessing further the models obtained in Table 2, using an independent set of wheat genotypes (elite and landraces) *n*, number of observations; NC, number of components; REP, relative error of prediction.

Experiment	Trait	NC	*n*	*R* ^2^	REP (%)	Bias (%)
CC-Mex2	SPAD	16	448	0.34	7.4	−3.5
L-Mex2	SPAD	16	270	0.44	6.6	−2.3
LS-Mex2	LMA	21	52	0.14	11.3	−3.3
LS-Mex2	N_area_	21	52	0.05	18.2	−5.5

**Fig. 6.**
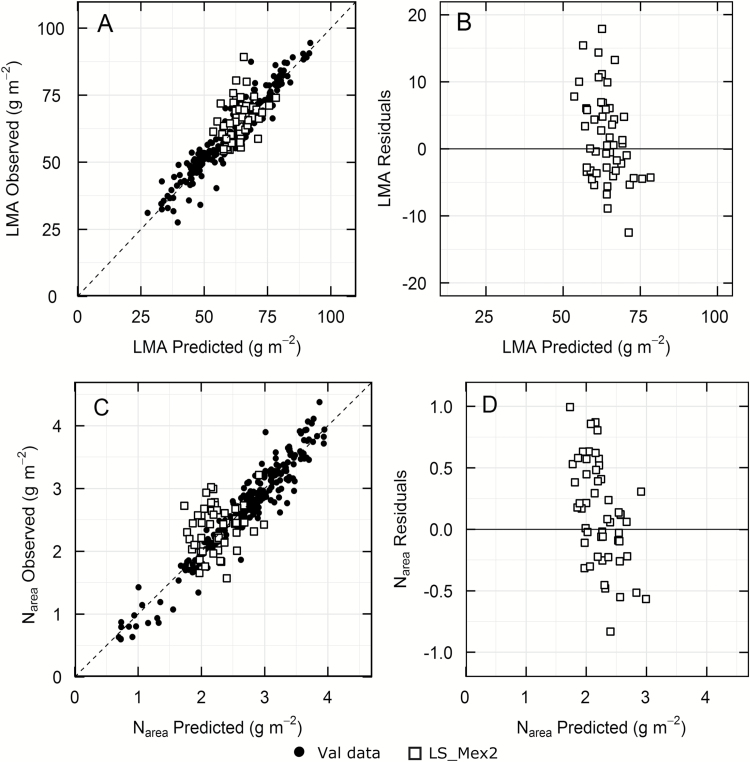
Comparison of predictions using the reflectance models for LMA (A) and N_area_ (C) against observed data for wheat landraces (LS-Mex2, open squares). The respective residuals are shown in (B) and (D). LS, *n*=52 and Val data, *n*=243. Closed circles are the validation data from Fig. 2A for N_area_ and Fig. 2B for LMA.

A subset of 21 wheat landraces and two elite wheats at a similar phenological stage were selected for a second measurement along with sampling to determine LMA and N_area_ (LS-Mex2). The model bias was −3.3% for LMA and −5.5% for N_area_. The relative error of prediction was 11.3% for LMA and 18.2% for N_area_, compared with 7% and 7.6%, respectively, observed for the validation data (Table 3). The residuals showed no underlying trend (Fig. 6B, D), but the ranges in the LS residuals were wider than the ranges in residuals observed for the original validation data (see Supplementary Fig. S6C, D).

### Prediction models using a narrower waveband

As not all spectrometers are able to measure both the visible and SWIR wavebands, we assessed the power of PLSR to predict parameters using only 400–900 nm reflectance values. Their performance was generally lower with the exception of SPAD (cf. Table 2). The *R*^2^ values for validation data were: N_area_, 0.83; LMA, 0.79; SPAD, 0.8; *V*_cmax_, 0.57; *J*, 0.56; *V*_cmax25_, 0.48; *V*_cmax25_/N_area_, 0.33. This indicates that significant information would be lost for the photosynthetic traits by omitting the SWIR1 and -2 bands, which would reduce the predictive power of the PLSR models.

## Discussion

The main objective of this experiment was to test if hyperspectral reflectance could be used to predict leaf nitrogen, LMA, and photosynthetic attributes in wheat. As hyperspectral reflectance can be measured relatively quickly, could this technique be used to screen for multiple traits and enable selection of wheat genotypes for photosynthetic traits? We based this work on a previous study conducted on aspen leaves (Serbin *et al.*, 2012). While the models developed to predict photosynthetic attributes for aspen were unsuccessful in wheat, we were able to develop new models for a variety of leaf traits. N_area_, LMA and SPAD were the traits with the highest coefficient of determination in the predictions. To assess their robustness, models were tested with previously unseen wheat genotypes. We also discuss the possibility of using calibration from other species to predict these traits.

### Predicting *V*_cmax_ and *J*

*V*_cmax_ and *J* are underlying biochemical traits that can be derived from CO_2_ response curves measured using gas exchange instruments. The two traits are usually estimated from the analysis of multiple measurements taken at different CO_2_ concentrations. The appeal of estimating *V*_cmax_ and *J* is that they are independent of stomatal conductance and represent the amount of Rubisco and components of the thylakoid electron transport chain, respectively (von Caemmerer, 2000). Measuring *A*–*C*_i_ curves to estimate *V*_cmax_ and *J* is slow. Each day the gas exchange system needs to be calibrated. Each leaf needs some time under the conditions imposed in the chamber of the gas exchange system before measurements begin, to allow stomata to open and metabolism to stabilize. Each *A*–*C*_i_ curve takes from 15 to 40 min, depending on the number of CO_2_ concentrations measured. Although faster approaches have been proposed, such as a rapid *A*–*C*_i_ curve (Stinziano *et al.*, 2017) or calculations using just one CO_2_ concentration (De Kauwe *et al.*, 2016), these methods have not been proven in high throughput screening of genetic material under field conditions.

By comparison with gas exchange measurements, hyperspectral reflectance using the ASD Field Spec is quick to calibrate before starting and it took from 15 to 50 s to measure a wheat leaf, depending on the settings. We found that a white reference calibration was not required before every measurement. From our experience in the field, a hyperspectral reflectance measurement was quicker to make than gas exchange measurements at a single CO_2_ concentration. Importantly, hyperspectral reflectance has the potential to predict as many parameters as there are calibrated models and can be used to measure hundreds of genotypes a day, as has been shown for maize (Yendrek *et al.*, 2017).

*V*_cmax_ for a leaf varies with temperature. To enable comparison between studies and because we were unable to maintain a constant leaf temperature over a day due to the natural fluctuations in ambient temperature (see Supplementary Fig. S1), we normalized *V*_cmax_ to 25 °C using revised Rubisco kinetics for wheat (*V*_cmax25_, Silva-Pérez *et al.*, 2017). A similar approach was used by Ainsworth *et al.* (2014) who measured leaf temperature immediately before reflectance measurements. When comparing observed parameter values derived from gas exchange measurements against those predicted from leaf reflectance, *V*_cmax_ and *J* both had a higher coefficient of determination than *V*_cmax25_ (*R*^2^=0.74 and 0.71, respectively, *vs* 0.62) (Fig. 3). This probably reflects the fact that the range in *V*_cmax_ (25–400 μmol CO_2_ m^−2^ s^−1^) was greater than for *V*_cmax25_ (23–280 μmol CO_2_ m^−2^ s^−1^). While the *R*^2^ value was lower for *V*_cmax25_ compared with *V*_cmax_, the relative error of prediction was also smaller (Table 2), suggesting that using *V*_cmax25_ is more accurate. The reflectance spectrum should be representative of the leaf composition, and hence the ‘capacity’ of the leaf, rather than the rate of the reaction *per se*. Another factor that could contribute to the disparity between *V*_cmax_ and *V*_cmax25_ models is if the temperature of the leaf during reflectance measurements affects the spectra. The leaf clip assembled as the factory default warms up due to the high photon flux from the internal lamp and this in turn warms the leaf during measurement. We did not observe a drift in spectral properties with sequential groups of scans that would have been associated with warming of the leaf. In most of our experiments, we used a mask with a gasket and measured the spectra within 9 s to reduce the impact of the high photon flux on leaf temperature. However, additional experiments to specifically look at the influence of leaf temperature on reflectance spectra are needed to assess this.

When the residuals from the PLS analysis of *V*_cmax_ and *J* were plotted against predicted values, no trends were apparent. However, when the residuals were plotted against observed values, positive trends were evident (see Supplementary Fig. S5), which indicates that factors not accounted for in the models are driving variation in the traits (Fox and Weisberg, 2011). A similar trend was evident in the prediction of *V*_cmax_ in maize (Yendrek *et al.*, 2017). Despite this limitation, the results show that leaf reflectance could be used to rank genotypes and select tails for *V*_cmax25_ from large populations. It would then be feasible to measure the smaller numbers of genotypes in the tails using gas exchange or other more laborious approaches for confirmation. As reflectance measurements are non-destructive, this facilitates making more measurements during the plant life cycle and on more leaves within plants, which could reduce error associated with variation in plant phenology and environmental effects when assessing genotypic variation of *V*_cmax25_ and *J*. In addition, reflectance using imaging spectroscopy has also shown promise for predicting *V*_cmax_ at the canopy level (Serbin *et al.*, 2015), which would provide an opportunity for canopy level high throughput estimation of photosynthetic parameters.

As with *V*_cmax_, *J* varies with temperature (June *et al.*, 2004; Silva-Pérez *et al.*, 2017). However, because the temperature response is known to vary as plants acclimate to their growth temperature (Bernacchi *et al.*, 2003), we chose not to assume a single temperature function across experiments to derive values for *J* at a common temperature. Caution is needed if using the current model for *J* when phenotyping. An improved model could be created if one had access to more calibration data collected at a single temperature.

*V*_cmax25_/N_area_ was calculated from the data obtained here as a possible estimate of photosynthetic efficiency (i.e. photosynthetic capacity per unit N invested at a leaf level). Interestingly, *V*_cmax25_/N_area_ when treated as a trait was predicted with a higher coefficient of determination directly than by predicting each component trait separately and then calculating the ratio. It may be that the N_area_ and *V*_cmax25_ had an additive effect in training the model more accurately. While the coefficient of determination was at the lower end of the traits examined, *V*_cmax25_ was normalized for temperature and then for leaf nitrogen, the *R*^2^ of 0.49 (Fig. 4) would still present an opportunity to explore genetic variation in this parameter. It presumably reflects variation in Rubisco kinetic properties and activation state (assumed to be constant), mesophyll conductance (as we assumed a common function for all genotypes) and N allocation at the leaf level.

### Predicting *A* and *g*_s_

*A* and *g*_s_ have been positively co-related with wheat yield (Fischer *et al.*, 1998) and are traits that need to be considered in selection of high yielding wheat genotypes. However, spot measurements of these parameters are sensitive to environmental effects.

Although light-saturated photosynthesis at ambient CO_2_ has previously been predicted in trees using leaf reflectance and transmittance (*R*^2^=0.74) (Doughty *et al.*, 2011), this is surprising since *g*_s_ can vary dynamically and its impact on the reflectance spectrum is unknown. When we examined our data, models predicting *A* and particularly *g*_s_ were weak (*A*, *R*^2^=0.49; *g*_s_, *R*^2^=0.34), with *g*_s_ having the greatest relative error of prediction (Table 2). Both traits can change quickly in response to clouds, fluctuating temperatures or in windy conditions, but the extent that this alters reflectance spectra has not yet been determined in wheat.

Other methods, such as infrared thermography, offer a better alternative to assess stomatal conductance in the canopy, as shown under water stress and salinity tolerance (Jones, 2007; Jones *et al.*, 2009; Sirault *et al.*, 2009; Munns *et al.*, 2010). Hand-held IR thermometry predicted *g*_s_ under irrigated field conditions (Amani *et al.*, 1996) and IR imaging increased accuracy and throughput (Tattaris *et al.*, 2016). The advantage of thermography is that many plots can be compared simultaneously when imaged from above. However, variation in canopy height can confound the interpretation (Rebetzke *et al.*, 2013). At the leaf level, the hand-held air-flow porometer (Fischer *et al.*, 1998; Rebetzke *et al.*, 2001) has been demonstrated to be a rapid and effective instrument to estimate *g*_s_.

### Predicting N_area_, LMA, and SPAD

Higher coefficients of determination and lower relative error of predictions were observed in the validation data for N_area_ and LMA compared with photosynthetic traits (Table 2). This agrees with measurements collected from multiple environments, nitrogen levels and different wheat species by Ecarnot *et al.*, (2013). The results from the current study are important since the plants evaluated were high yielding wheat and triticale, many of which are currently used by farmers around the world.

SPAD was used in this study as a ‘trait’ because it is quick and easy to deploy in the field and could be compared with predictions derived from hyperspectral reflectance. During the data validation (Fig. 2E; *R*^2^=0.82) and in experiments with different wheat populations (Fig. 5), strong positive correlations were observed between measured and predicted SPAD values, in agreement with biochemical extraction (Doughty *et al.*, 2011) or from the chlorophyll normalized difference index (Dillen *et al.*, 2012).

### Predicting traits from reflectance measured in diverse sets of wheat genotypes

Models derived from aspen and cotton leaves were able to predict leaf nitrogen concentration and LMA from reflectance measurements on soybean (Ainsworth *et al.*, 2014), suggesting that these models are robust across a range of species. However, the model predicting LMA with 22 wavelengths and an intercept for aspen trees (Serbin *et al.*, 2012) gave variable results for wheat (see Supplementary Fig. S7). While most of the experiments could be predicted with the aspen LMA model, data measured in the field in Mexico could not. The possibility of developing a robust model to predict LMA across diverse species is appealing and published results show some promise (Heckmann *et al.*, 2017). Here we tested our models with wheat genotypes that had not been used to develop the models for SPAD, LMA and N_area_ (Figs 5 and 6). The relative error of prediction increased for this material, but as more calibration data become available, one would expect that the predictive ability for LMA would improve.

Models for leaf nitrogen concentration and *V*_cmax_ (see Supplementary Fig. S7) from aspen (Serbin *et al.*, 2012) did not predict these traits in wheat. In this study, a mask (Supplementary Fig. S3) was used in the leaf-clip of the ASD Field Spec to narrow the aperture so that all the wheat leaves filled the field of view. It is possible that this change in measurement geometry affected the comparison. Transferability of carbon:nitrogen ratio models between two Brassicaceae genera was poor and the performance of photosynthetic trait models was less accurate when applied to a species that had not been used to construct the model (Heckmann *et al.*, 2017). Thus, each model needs to be validated for the species of interest.

In general, the predictions obtained in this study for wheat were higher or within the range of *R*^2^ for predictions of similar traits that have been reported for other species using hyperspectral leaf reflectance (Table 4). Validations for different species shown in Table 4 indicate which traits can be well predicted using hyperspectral leaf reflectance and whether they apply across species or not. Variation in kinetic parameters for *V*_cmax_ between species may not be evident in the reflectance spectra. In contrast, LMA or leaf nitrogen might be more robust traits that can be predicted from a single reflectance model applied to different species.

**Table 4.** Comparison of the coefficients of determination (R^2^) for leaf traits taken from publications and this paper *A*_400_, *A*_1500_ and *A*_2000_, CO_2_ assimilation rate measured at 400, 1500 and 2000 μmol CO_2_ mol^−1^ inlet CO_2_, respectively. IS, initial slope of the *A*–*C*_i_ response curve.

Plant material and source	*V*_cmax_/*V*_cmax25_/IS	*J*	LMA/SLA	N_mass_/N_area_	Chlorophyll/SPAD	*A*_400_/*A*_1500_/*A*_2000_
159 tropical plants (Doughty *et al.*, 2011)	*V*_cmax_ 0.39	0.52	LMA 0.9	N_mass_ 0.83	Chlorophyll 0.66 (Chl *a*) Chlorophyll 0.67 (Chl *b*)	*A*_400_ 0.74 *A*_1500_ 0.47
Aspen, cotton (Serbin *et al.*, 2012)	*V*_cmax_ 0.89	0.93	LMA 0.95	N_mass_ 0.89		
Wheat (Ecarnot *et al.*, 2013)			LMA 0.94	N_mass_ 0.94		
Soybean (Ainsworth *et al.*, 2014)	*V*_cmax25_ 0.88					
Maize (Yendrek *et al.*, 2017)	*V*_cmax_ 0.65		SLA 0.68	N_mass_ 0.96	Chlorophyll 0.85	
*Brassica* *Moricandia* Maize (Heckmann *et al.*, 2017)	IS 0.55 IS 0.59 IS 0.54					*A*_2000_ 0.49 *A*_2000_ 0.37 *A*_2000_ 0.62
This study Wheat/triticale	*V*_cmax_ 0.74 *V*_cmax25_ 0.62	0.71	LMA 0.89	N_mass_ 0.7 N_area_ 0.93	SPAD 0.81	*A*_400_ 0.49

### Training set size and source of variation

Each hyperspectral reflectance generates 2000 values that are used to calculate each trait. PLSR solves the problem of dimensionality and multicolinearity and the issue of overfitting is dealt with by using the lowest PRESS or RMSE to determine the number of components to be used (Geladi and Kowalski, 1986). However, the question of how many observations are needed to train the model remains. In maize, 80% of the observations were used to train the model (Yendrek *et al.*, 2017). In *Brassica* a subset size of 90 observations and in maize 30 observations resulted in the lowest RMSE (Heckmann *et al.*, 2017). With wheat, we used about 55% of the observed data for training: 282 measurements were used to build the model to predict LMA, N_area_ and SPAD. Ecarnot *et al.*, (2013) used reflectance to predict LMA and N_area_, using a calibration obtained from a diverse collection of wheats measured under multiple conditions and environments (176–601 leaves). The calibration for aspen required 78 observations (Serbin *et al.*, 2012). In both of these studies, environmental treatment was a stronger driver of variation than genetic variation and the wide range of values improved the fit. Further analyses comparing the impact of training set size and range in the spectral data used to construct the models are required.

### Advantages of using hyperspectral reflectance

The data presented here suggest that the models we obtained provide robust estimates for six different traits from a single hyperspectral reflectance measurement. Approximately 100 plants could be measured per hour in the field using the hyperspectral reflectance technique described in this study (two people measuring one plant per 6 m-long plot in the field). Screening leaf physiological and biochemical parameters using this approach will enable larger populations to be analysed for photosynthetic characters that can be combined with molecular markers and genomic sequences to find regions in the plant genome related to variation in photosynthetic performance (quantitative trait loci).

### Concluding remarks

We have demonstrated the utility of leaf hyperspectral reflectance modelling to screen large wheat germplasm sets for *V*_cmax25_, *J*, SPAD, LMA, N_area_, and *V*_cmax25_/N_area_, a range of photosynthetic traits not easily derived in high throughput from other methodologies. This will enable wheat researchers and breeders to rapidly identify genetic variation in germplasm for crossing, genetic mapping and identification of material for more detailed mechanistic analysis.

## Supplementary data

Supplementary data are available at *JXB* online.

Fig. S1. Meteorological conditions in Obregon, Mexico and Ginninderra, Australia.

Fig. S2. Histogram of the days after flowering (DAF) when the landraces were surveyed for reflectance.

Fig. S3. Measuring reflectance with the leaf clip, showing leaf orientation and mask.

Fig. S4. Regression coefficients for PLSR models.

Fig. S5. Residuals from Figs 2–4 plotted against observed data.

Fig. S6. Density plots of residuals of the predictions.

Fig. S7. Validation of predictions using reflectance with the coefficients from Serbin *et al.*, 2012 against observed data for wheat.

Table S1. Inlet CO_2_ concentrations used in each experiment to measure CO_2_ response curves.

Table S2. Training data and test data from experiments used in the PLSR model.

Supplementary Tables S1-S2 and Figures S1-S7
