# Supplementary material for: Hyperspectral reflectance as a tool to measure biochemical and physiological traits in wheat
Source: J Exp Bot. 2017 Dec 22;69(3):483–96. doi: 10.1093/jxb/erx421 (PMC5853784; doi:10.1093/jxb/erx421)
Supplement: Supplementary Tables S1-S2 and Figures S1-S7 [file erx421_suppl_supplementary_tables_s1-s2_figures_s1-s7.pdf]

Hyperspectral reflectance as a tool to measure biochemical and physiological traits in wheat

SUPPLEMENTARY TABLES

Table S1. Inlet CO_2 concentrations used in each experiment to measure CO_2 response curves

Set of genotype/Experiment	Inlet CO_2 (μmol CO_2 mol^{-1})
**EVA-Aus1**	50, 100, 250, 400, 800
**BYPB-Aus2**	50, 100, 250, 400, 800
**BYPB-Aus3**	50, 100, 250, 400, 800
**EVA and CA-Aus3**	50, 150, 250, 400, 600, 800, 1200
**CA-Mex1**	50, 100, 250, 400, 600, 800

Table S2. Training data and test data from experiments used in the PLSR model. Glasshouse (GH). Fertilized plants (+N), not fertilized (-N). Repetition (Rep). Number of genotypes used (Gen). Experiment (Exp).

Exp (Gen)	Training data	Test data
**Aus1** (16)	GH(+N) Rep 1 & 2<br>GH(-N) Rep 1 & 2	GH(+N) Rep 3<br>GH(-N) Rep 3
**Aus2** (30)	GH(+N) Rep 1<br>GH(-N) Rep 1	GH(+N) Rep 2<br>GH(-N) Rep 2
**Aus3** (2)<br>**Aus3** (26)<br>**Aus3** (20)	Field EVA Rep 1 & 2<br>Field BYPB Rep 1 & 2<br>Field CA Rep 1 & 2	Field EVA Rep 3 & 4<br>Field BYPB Rep 3 & 4<br>Field CA Rep 3 & 4
**Mex1** (30)<br>**Mex1** (30)*	Field CA Rep 1 & 2<br>Field CB Rep 1 & 2*	Field CA Rep 3<br>Field CB Rep 3*

***CB_Mex** measurements were only used for SPAD predictions. Other traits were not considered for these set of genotypes.

SUPPLEMENTARY FIGURES

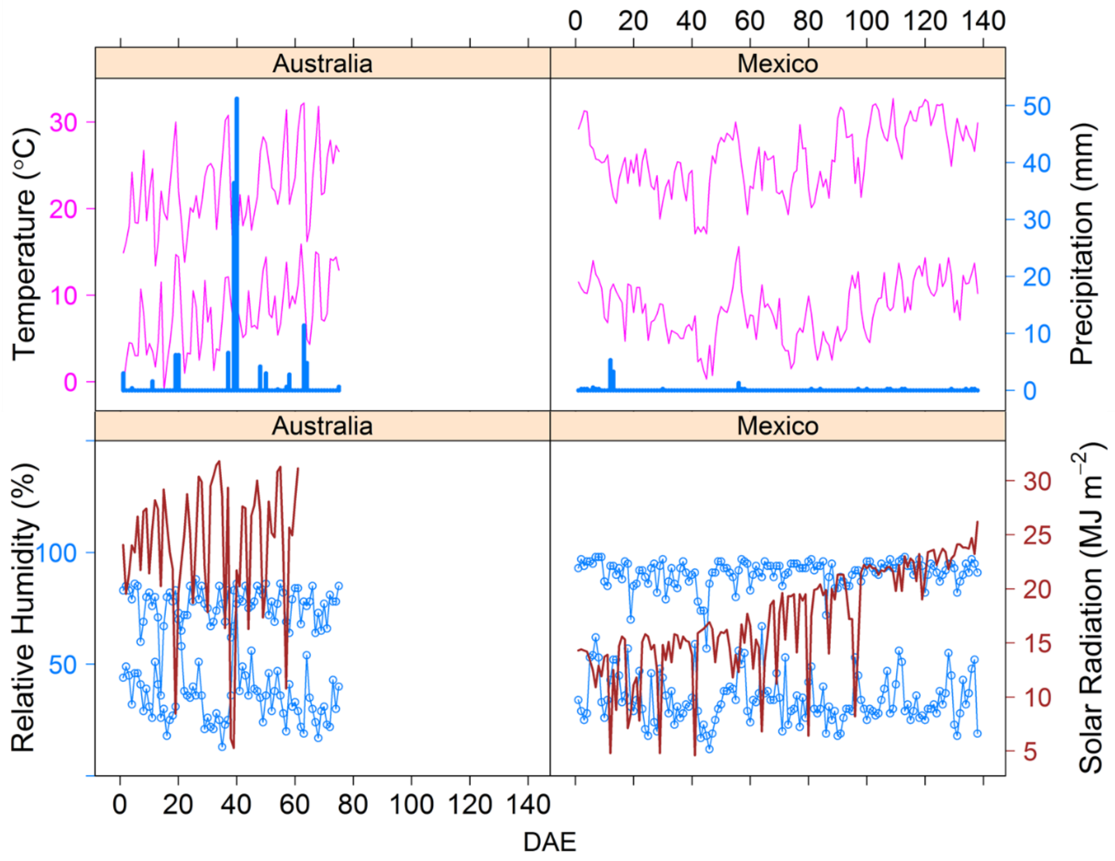

Figure S1. Meteorological conditions in Obregon, Mexico and Ginninderra, Australia. Daily observations from days after emergence (DAE) during measurements in Australia (1-75 DAE) and in Mexico (1-138 DAE). Minimum and high temperature in pink, precipitation in blue bars, minimum and maximum humidity (white circles) and solar radiation in red. Spray irrigation was carried out in Australia on DAE 24, 56 and 77, with approximately 25mm of water being applied. Furrow irrigation was carried out in Mexico at sowing and on DAE 39, 61, 82 and 103, providing 100 mm of water per event.

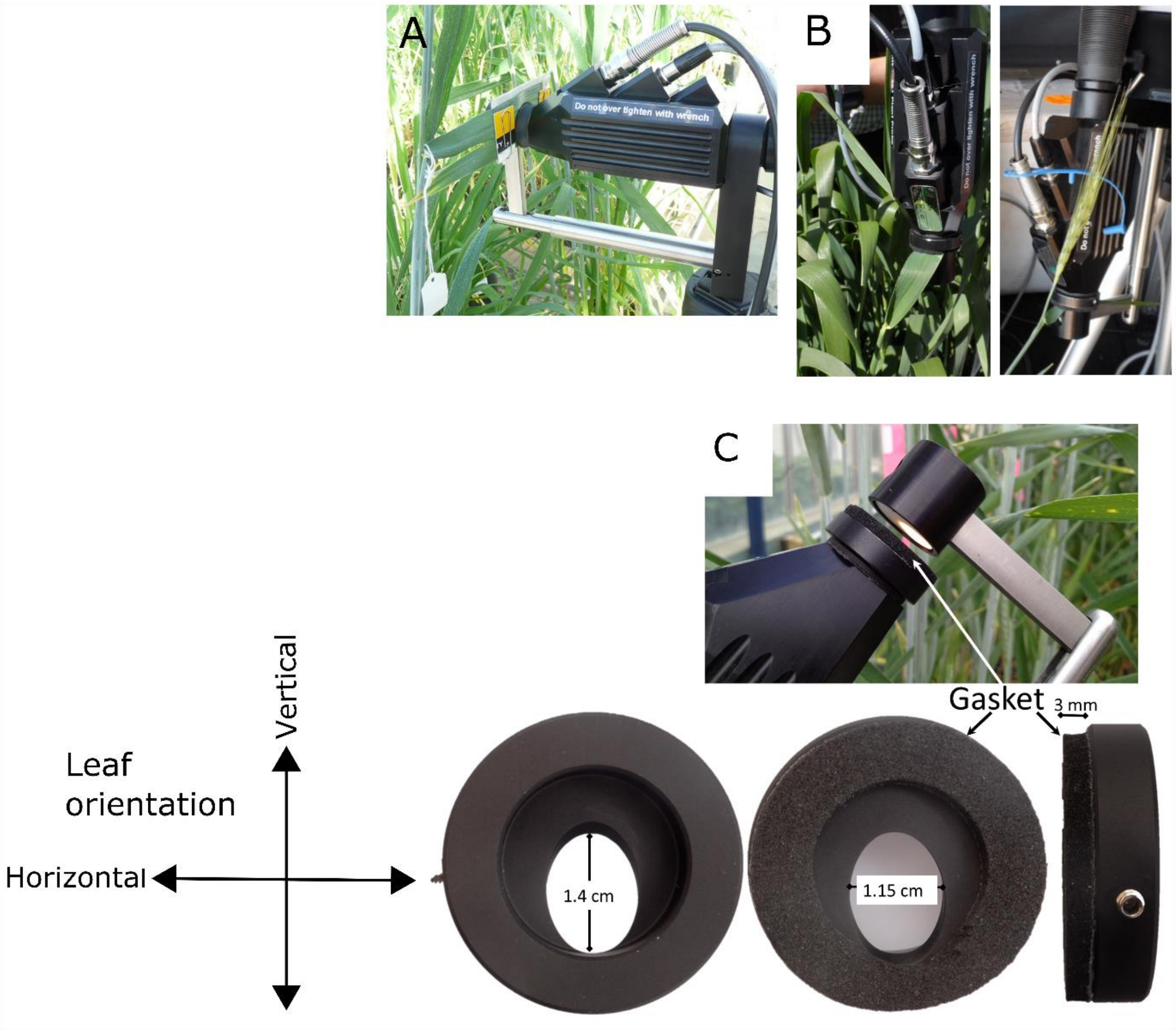

Figure S2. Measuring reflectance with the leaf clip. A) two leaves were placed horizontally with respect to the leaf clip. B) With the mask in place, placing one leaf vertically covered the field of view. C) Detail of the mask and gasket reducing the field of view. Vertical means in relation to the leaf clip. The long axis of the oval hole in the mask aligns with the shape from the light source.

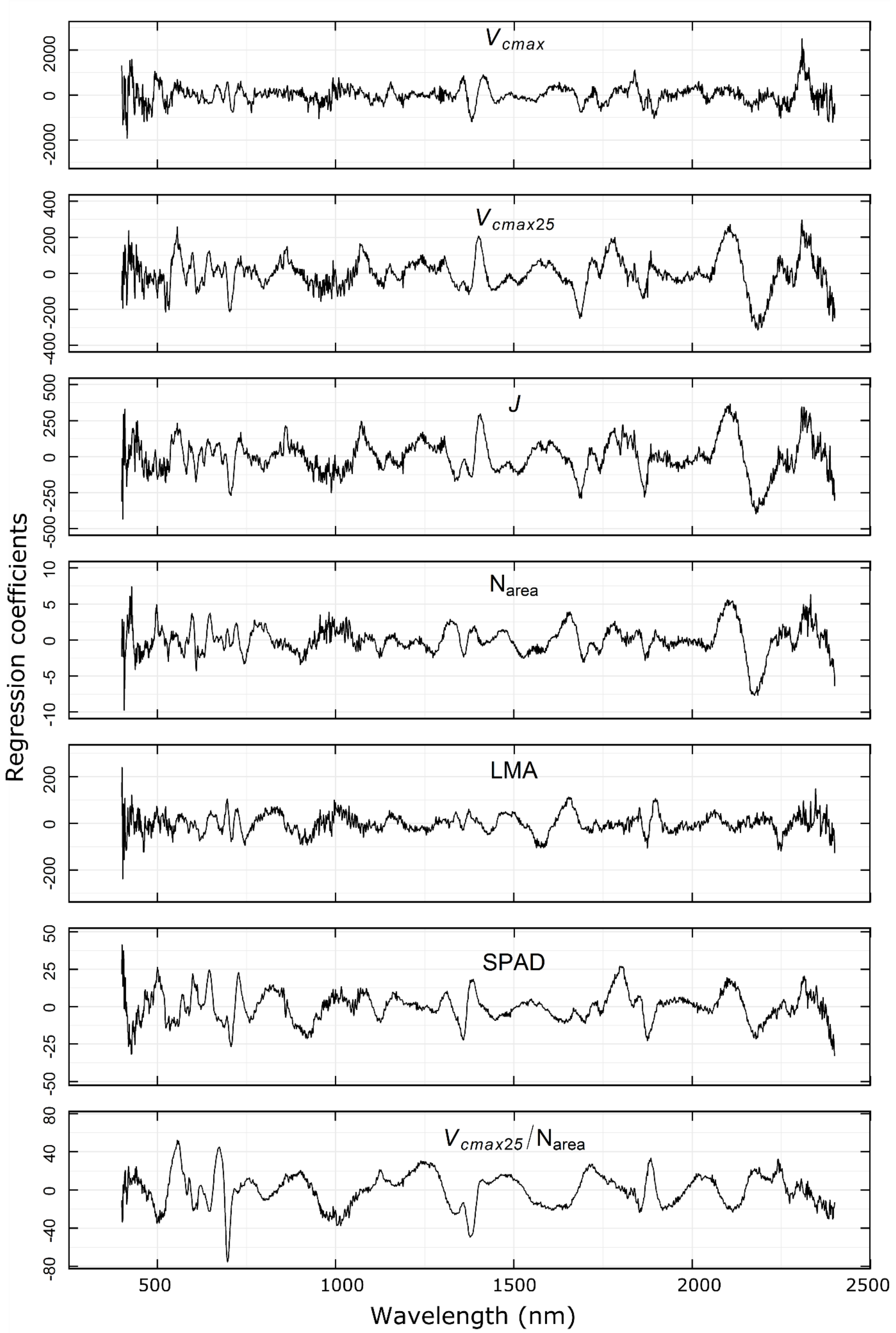

Figure S3. Regression coefficients for V_{cmax}, V_{cmax25}, J, N_{area}, LMA, SPAD and V_{cmax25}/N_{area}.

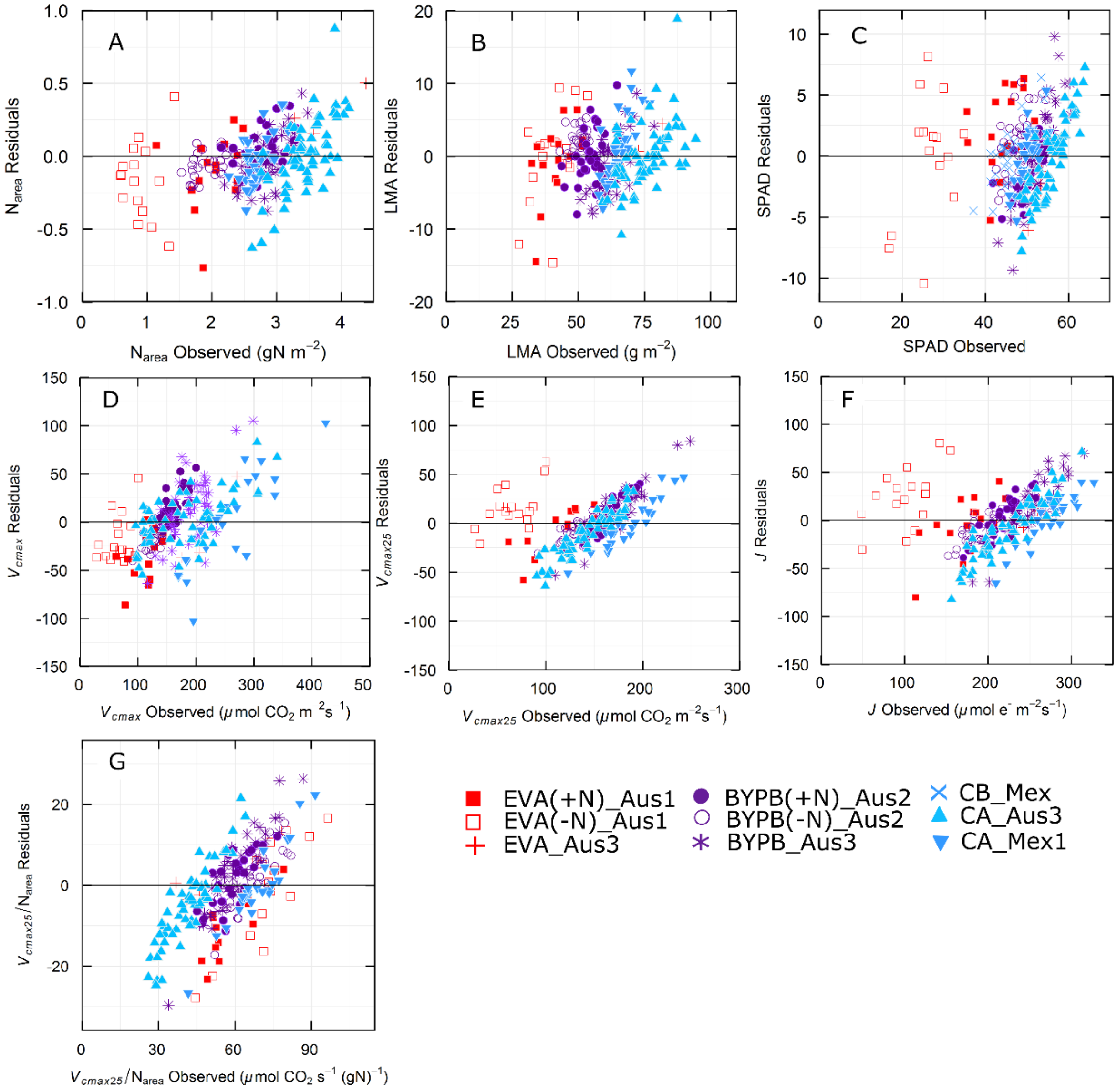

Figure S4. Residuals from Fig. 2, 3 and 4 plotted against observed data of A) N_{area}, B) LMA, C) SPAD, D) V_{cmax}, E) V_{cmax25}, F) J and V_{cmax25}/N_{area}

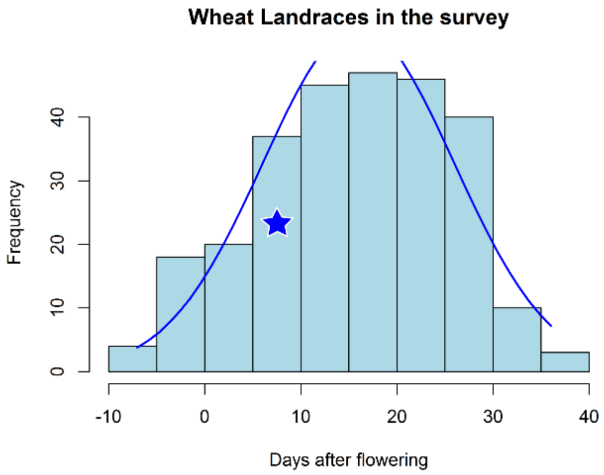

Figure S5. Histogram of the days after flowering (DAF) when the landraces were surveyed for reflectance, the star marks 5-10 DAF.

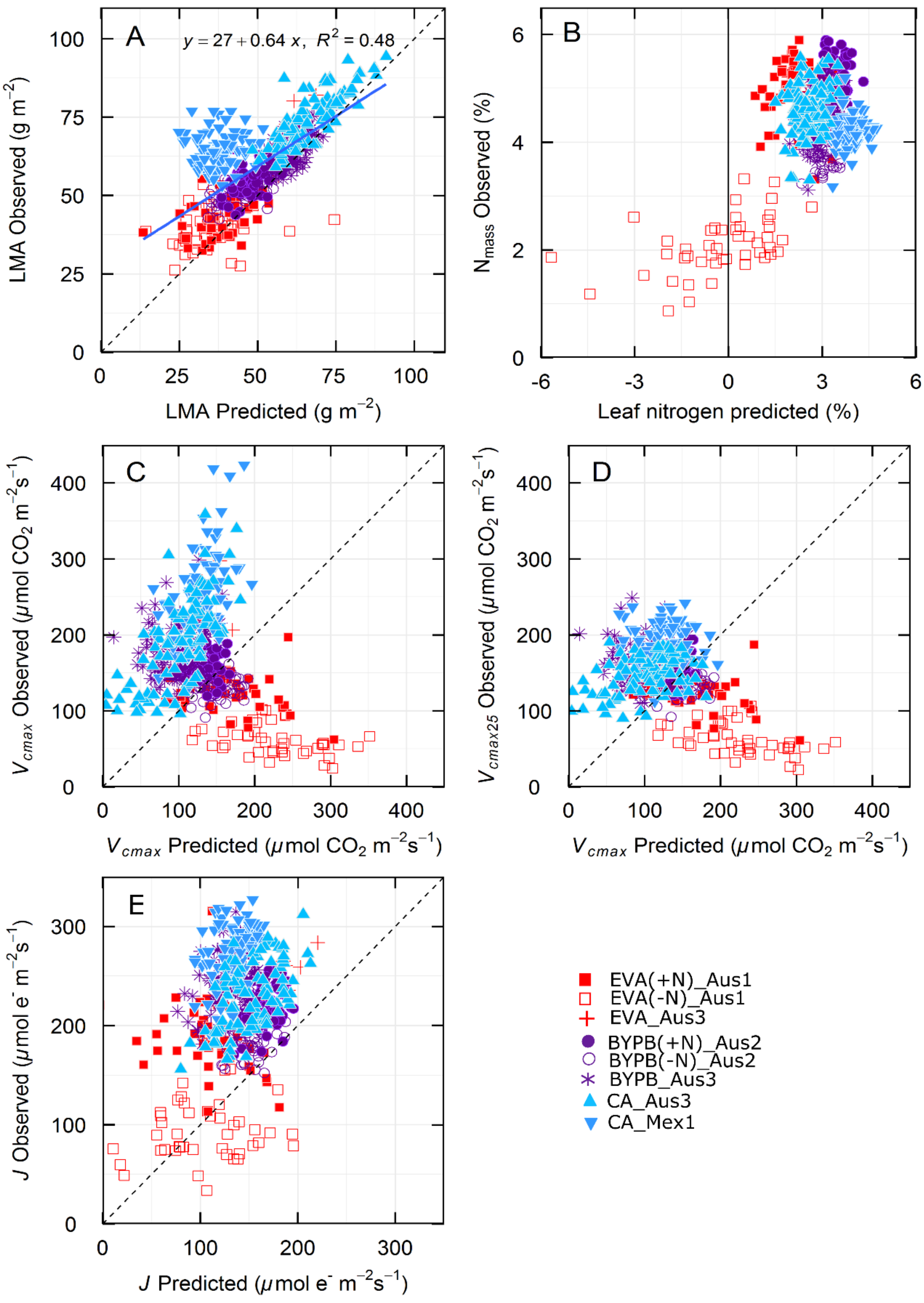

Figure S6. Validation of predictions using reflectance with the coefficients from Serbin *et al*., 2012 against observed data for wheat A) LMA (n=525), B) N % (n=525), C) V_{cmax}, D) V_{cmax25} (n=488) and E) J (n=488).

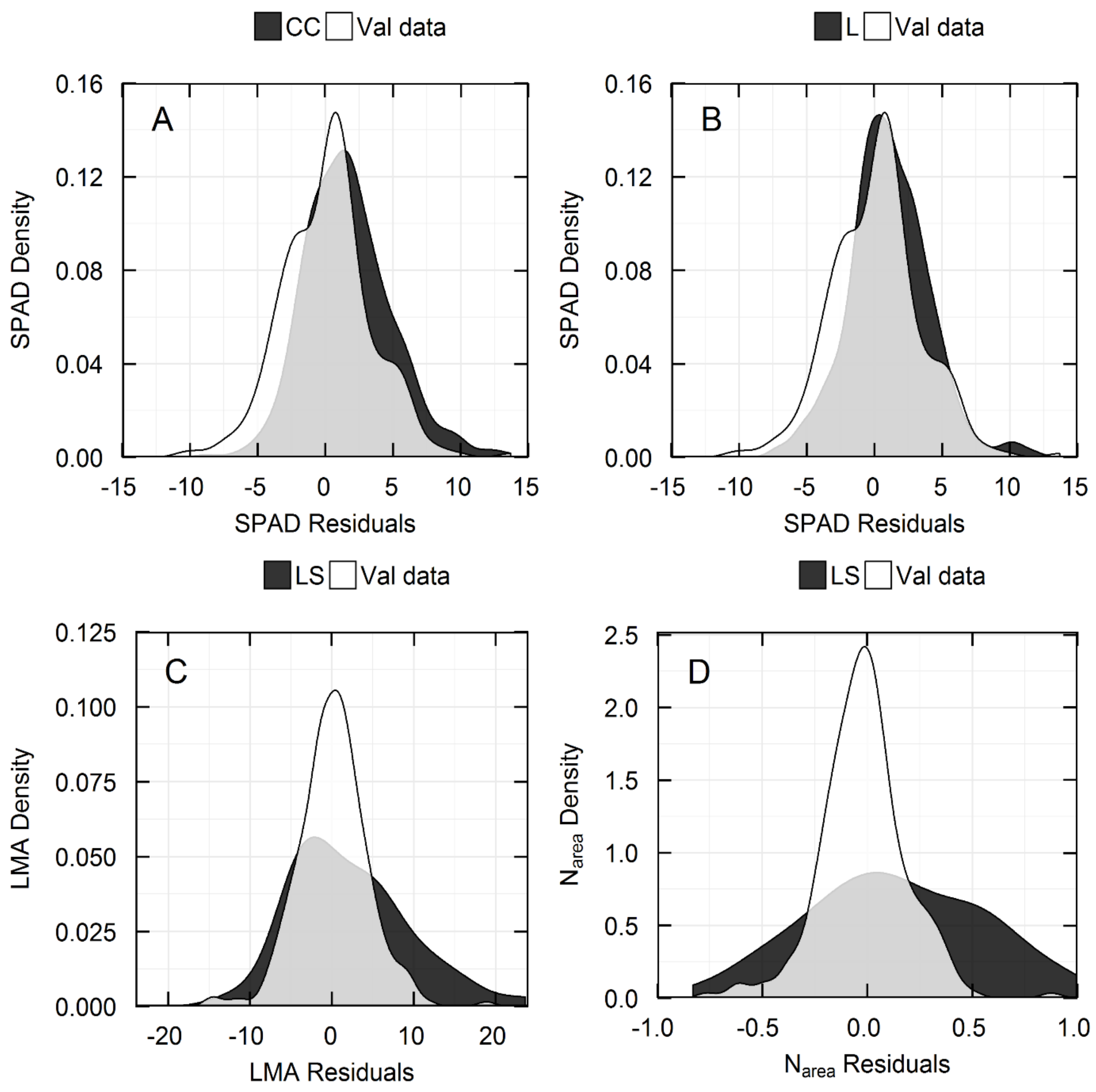

Figure S7. Density plots of residuals of the predictions A) and B) for SPAD (CC, n=448, L, n=270 and Val data, n=272), C) and D) LMA and N_{area} (LS, n=52 and Val data, n=243).
